# Geography Influences Dietary Intake, Physical Activity and Weight Status of Adolescents

**DOI:** 10.1155/2012/816834

**Published:** 2012-05-23

**Authors:** Shauna M. Downs, Shawn N. Fraser, Kate E. Storey, Laura E. Forbes, John C. Spence, Ronald C. Plotnikoff, Kim D. Raine, Rhona M. Hanning, Linda J. McCargar

**Affiliations:** ^1^Department of Agricultural, Food & Nutritional Science, 2-021D Li Ka Shing Centre, University of Alberta, Edmonton, AB, Canada T6G 2E1; ^2^Centre for Nursing and Health Studies, Athabasca University, Athabasca, AB, Canada T9S 3A3; ^3^Department of Public Health Sciences, School of Public Health, University of Alberta, Edmonton, AB, Canada T6G 1C9; ^4^Faculty of Physical Education and Recreation, University of Alberta, Edmonton, AB, Canada T6G 2H9; ^5^School of Education, Faculty of Education and Arts, University of Newcastle, Newcastle, NSW 2308, Australia; ^6^Centre for Health Promotion Studies, School of Public Health, University of Alberta, Edmonton, AB, Canada T6G 1C9; ^7^Health Studies and Gerontology, Faculty of Applied Health Sciences, University of Waterloo, Waterloo, ON, Canada N2L 3G1

## Abstract

*Purpose*. The purpose of this study was to assess rural and urban differences in the dietary intakes, physical activity levels and weight status of a large sample of Canadian youth in both 2005 and 2008. *Materials and Methods*. A cross-sectional study of rural and urban adolescents (*n* = 10, 023) in Alberta was conducted in both 2005 and 2008 using a web-based survey. *Results*. There was an overall positive change in nutrient intakes between 2005 and 2008; however, rural residents generally had a poorer nutrient profile than urban residents (*P* < .001). They consumed less fibre and a greater percent energy from saturated fat. The mean physical activity scores increased among rural youth between 2005 and 2008 (*P* < .001), while remaining unchanged among urban youth. Residence was significantly related to weight status in 2005 (*P* = .017), but not in 2008. *Conclusion*. Although there were small improvements in nutrient intakes from 2005 to 2008, several differences in the lifestyle behaviours of adolescents living in rural and urban areas were found. The results of this study emphasize the importance of making policy and program recommendations to support healthy lifestyle behaviours within the context of the environments in which adolescents live.

## 1. Introduction

Overweight and obesity rates of adolescents in Canada, the United States, and worldwide are prevalent and increasing [1–5]. The rise in overweight and obesity levels may be due to changes in the lifestyles and environments in which youth live. There are various determinants that influence the risk of overweight and obesity in youth, including geographical region [6]. Socioeconomic status (SES), opportunities for physical activity, and differences in food availability have been proposed as explanations for the disparities in disease risk between individuals living in rural and urban areas [7–10].

Rural and urban differences in the rates of overweight and obesity in Canadian youth have been reported [11–14]; however, these differences have not been consistent. Findings from the 2004 Canadian Community Health Survey (CCHS) revealed that Alberta was the only province in Canada to have lower rates of overweight and obesity (combined) among youth living in urban centres, compared to rural dwellings; there were no significant differences in the weight status of children living in rural and urban areas elsewhere in Canada [14]. Although geographical variations in the overweight and obesity prevalence of youth in Alberta have been reported, there is little evidence to help explain this difference. A Canadian study that included Alberta adolescents found higher intakes of calcium and milk products and a trend towards higher consumption of high fat/sugar foods in rural students, compared to urban students [15]. However, physical activity levels in the same sample of adolescents were similar in both rural and urban students [16]. As part of comprehensive school health programs, healthy eating and active living policies need to be developed, implemented and tailored to the school-specific context [17]. It is therefore necessary to fully understand the relationship among school environments, dietary intake, physical activity levels, and weight status. In recent years, schools have become more aware of their role in shaping the health behaviours of their students; thus the purpose of this study was to assess rural and urban differences in the dietary intakes, physical activity levels and weight status of Alberta youth in both 2005 and 2008. We hypothesized that (1) there would be an improvement in dietary intakes, physical activity levels and weight status from 2005 to 2008 and (2) that rural students would differ from urban students in health behaviours and weight status.

## 2. Materials and Methods

### 2.1. Subjects

A previously validated Web-Survey of Physical Activity and Nutrition (Web-SPAN) was used to obtain information regarding the dietary intakes, physical activity levels and weight status of Alberta youth [18]. The anonymous, self-administered, web-based survey was completed by students in grades 7 through 10 in 2005 and in another similar cohort in 2008. In both 2005 and 2008, the data were collected throughout the year, with the exception of July and August (when the schools were closed). A detailed description of the survey is reported elsewhere [18]. Recruitment techniques differed slightly in 2005 compared to 2008. In 2005, all Alberta school boards were contacted and invited to participate in the study. Of the school boards that agreed to participate, an average of seven schools were randomly selected from each school board. However, in 2008, schools were randomly selected to participate from a list of all Alberta schools that met the inclusion criteria. The school boards of these schools were then contacted to gain study approval. Principals then indicated which classes would participate in the survey depending on the school configuration. Of the selected classrooms, all students were invited to participate in the survey. Students completed the survey during class time under teacher supervision.

School boards and schools were initially contacted by mail and later by telephone to request participation. Superintendents and school principals provided verbal consent for the study. Parents and students received information letters outlining the study details. Parents of the participating students signed written consent forms and students provided assent after logging on to the survey. Students were able to terminate the survey at any time and were not required to answer every question. Outliers were identified using predetermined criteria from the 2005 survey; extreme outliers were identified using SPSS statistical software (version 19.0.0.1; 2010, SPSS Inc, Chicago, IL) and were removed from the database [18]. Overall, students who signed onto the survey but did not complete any part of the survey (2005: *n* = 9, 2008: *n* = 26), and extreme outliers of total energy intake (2005: *n* = 24, 2008: *n* = 33), physical activity (2005: *n* = 14, 2008: *n* = 19), and BMI (2005: *n* = 34, 2008: *n* = 33) were excluded from all analyses based on outlier analysis. School postal codes were used to define them as being either urban or rural and to determine median neighbourhood income. Urban schools were defined as those located in areas identified by Statistics Canada as Census Metropolitan Areas or Census Agglomerations and Rural Small Town areas were classified as rural [19]. Median neighbourhood income was used as a measure of SES. Ethics approval for this study was obtained from the Human Research Ethics Board in the Faculty of Agricultural, Life and Environmental Sciences at the University of Alberta and by the Cooperative Activities Program in the Faculty of Education at the University of Alberta.

In 2005, of the 363 schools that were contacted, the final participation was 4932 students from 136 schools representing 44 school boards (37% school response rate; 75% school board response rate). In 2008, of the 271 schools that were approached to participate, the final participation was 5091 students from 109 schools representing 37 schools boards (40% school response rate; 64% school board response rate). Overall, 10,023 youth from 2005 and 2008 participated in the study. The mean age of students was comparable in 2005 (13.6 ± SD 1.2 yr) and 2008 (13.3 ± SD 1.5 yr).

### 2.2. Dietary Intakes

The web-survey assessed dietary intake using a validated 24-hour dietary recall [15, 18]. Students were asked about food consumption on the previous day. In order to avoid weekend intakes, which tend to be more variable and unusual than weekdays, teachers were asked to have students complete the survey Tuesday through Friday. Diet quality was based on meeting the minimum serving recommendations for Eating Well with Canada's Food Guide food groups [20]. Using this food-based measure of diet quality, participants were classified as having poor diet quality if they met 0-1 food group recommendations, average diet quality if they met 2-3 recommendations and superior diet quality if they met the recommendations for all four foods groups. A detailed rationale for the use of this measure is reported elsewhere [18]. Food Processor SQL for Windows package version 7.9 (ESHA Research, Salem, OR, USA) and the Canadian Nutrient File (2001b) were used to obtain the nutrient composition of the foods and beverages consumed.

### 2.3. Physical Activity

The validated Physical Activity Questionnaire for Older Children (PAQ-C) was used to assess self-reported physical activity levels over a 7-day period [21]. PAQ-C scores range from 1 (no physical activity) to 5 (high amount of physical activity); a score of 3 is considered a moderate activity level. 

### 2.4. Weight Status

Body mass index (BMI) was calculated using self-reported heights and weights. The International Obesity Task Force (IOTF) cut-offs were used to determine weight status [22].

### 2.5. Statistical Analyses

Differences between geographical location (rural and urban) and Web-SPAN year (2005 and 2008) were analysed with linear mixed modelling (LMM) and estimated with restricted maximum likelihood estimation (REML) controlling for SES, age and sex. An intraclass correlation coefficient (ICC) was calculated for each dependent variable using an intercepts only model to assess the extent of variability in the dependent variable between schools and to account for clustering. Following a significant interaction, simple effects analyses were conducted. Differences were examined between geographic region within each year and differences between each year within urban and then rural levels. Chi-squared tests were used to examine differences between categorical variables. Data were examined for major violations of the assumptions of LMM. Because students were not required to answer every question on the web-survey, sample sizes vary throughout. All statistical analyses were performed using SPSS (version 19.0.0.1; 2010, SPSS Inc, Chicago, IL).

## 3. Results

The ICCs for nutritional variables ranged from  .016 for percent energy from saturated fat to  .032 for grams of fibre and total energy. These low ICCs suggest that school level had little impact on estimates. The ICC for physical activity was  .08 indicating a small impact of school on the variability in PAQ-C scores. Despite these low ICCs, LMM controlling for clustering at the school level was conducted since even small ICCs can lead to underestimated standard errors, and therefore, an inflated type 1 error rate.

### 3.1. Dietary Intakes

Energy and nutrient intakes, adjusted for age and sex, of rural and urban youth in 2005 and 2008 are reported in Table 1. An REML LMM was performed for each of the five dependent variables controlling for SES, age and sex as fixed effects: total energy, percent energy from fat, saturated fat, carbohydrate, protein and grams of fibre. Web-SPAN year (2005 and 2008) and residence (rural and urban) were the fixed (between subjects) effects.

 Changes in nutrient intakes were noted from 2005 to 2008, with increases in grams of fibre and percent energy from protein and a reduction in total energy and percent energy from fat. Rural residents had a generally poorer nutrient profile than urban residents (*P* < .001). Specifically, rural students consumed a higher percentage of energy from saturated fat than urban students and rural students consumed fewer grams of fibre than urban students. A significant interaction between year and residence (*P* = .042) showed that rural and urban youth differed in changes from 2005 to 2008 in terms of percent energy from saturated fat. Specifically, urban youth decreased the percent of energy from saturated fat from 2005 to 2008 and by 2008, urban youth consumed a lower percent of energy from saturated fat than rural youth.

Table 2 describes diet quality based on year and residence. A chi-square test showed that residence was significantly related to diet quality in 2005 (*P* = .002) but not in 2008 (*P* > .10). The source of this difference was a larger proportion of rural children in the poor diet category and a smaller proportion in the average diet quality category in 2005. Results also showed that urban children had a larger proportion of children in the poor diet quality category and fewer in the average diet quality category in 2008 compared to 2005.

### 3.2. Physical Activity

After controlling for SES, age, and sex, an REML LMM controlling for school level clustering revealed a significant main effect for year (*P* = .024), but not residence (*P* > .10), and a significant interaction between year and residence (*P* = .033). In terms of the interaction, the mean PAQ-C score among rural youth increased from 2005 to 2008, and by 2008 rural youth were more active than urban youth (Table 2).

### 3.3. Weight Status

Table 3 describes weight status by year and residence. A chi-square test showed residence was significantly related to weight status (*P* = .004) where there were more normal weight children in urban areas than rural areas, and there were more overweight and obese children in rural than urban areas. These differences reflect a larger number of urban children in the normal weight category and fewer in the overweight category than rural children in 2005. There were no differences between urban and rural children's weight status in 2008. There was also a difference in weight status in terms of year (*P* = .001). Specifically, there were more normal weight children and fewer obese children in 2008 compared to 2005. The main source of these differences came from an increase in the proportion of those in the normal weight category among rural children from 2005 to 2008 and a decrease in the proportion of obese children from 2005 to 2008 among urban children.

## 4. Discussion

Over the past few years, as child and adolescent obesity rates have increased, the school environment has become an optimal location for policy and program interventions to help attenuate the rise of unhealthy weights. Overall, students consume approximately one third of their total daily energy intake at schools, although the quality of that energy can be quite variable [23, 24]. In some cases, schools may present a negative nutrition and physical activity environment [25]. Comprehensive school health approaches to improving diet and physical activity levels in children and youth are required [17]. This study found a small overall improvement in diets from 2005 to 2008; however, there were differences in the dietary intakes, diet quality, physical activity levels and weight status of Alberta adolescents attending rural and urban schools.

In recent years, school nutrition policies have become increasingly popular. In the United States, many school districts were required to develop nutrition policies in 2006 [25]. Although Alberta schools are not required to form nutrition policies, in 2005 under the Integrated Pan-Canadian Healthy Living Strategy, health ministers committed to develop school nutrition standards and healthy eating programs [26]. School nutrition has arguably become a more prominent focus of schools since 2005. It is, therefore, important to highlight the small overall improvement in nutrients and the decrease in energy intakes from 2005 to 2008 that was shown in this study. It is important to note that although the differences in nutrients may appear small, they are important given that early nutrition effects key risk factors for chronic disease [27]. Decreases in saturated fat will likely benefit youth over time [28], particularly since Alberta youth currently exceed the saturated fat recommendations. 

It is possible that school nutrition initiatives have positively influenced the diets of Alberta adolescents. Nevertheless, rural students consumed more saturated fat and less fibre than urban students. Moreover, rural youth had lower diet quality than urban students in 2005. This suggests that rural students may be consuming less nutritious foods than their urban counterparts. Similarly, previous studies conducted in Alberta have found a greater consumption of energy-dense foods in rural areas [15, 29]. These differences in dietary intakes can likely be attributed to the rural food environment. Rural stores tend to have higher food prices, lower quality fresh foods, and less variety than urban stores [30–32]. It is possible that the availability of food in rural environments may have contributed to the higher intakes of saturated fat and the lower diet quality in 2005. It is particularly important for rural schools to have sufficient access to healthy, competitively priced food. Although dependent on climate and availability, school food contracts with local producers and farmers in rural areas may be an effective strategy to improve access to healthy food in rural areas potentially resulting in intakes of healthier food [33].

Over the three years, between 2005 and 2008, physical activity levels increased in rural students and remained the same among urban residents. A recent U.S. study that used the PAQ-C to measure physical activity found urban children were less active than children living in rural areas and small cities [34]. It has been suggested that daily, quality, safe physical education and activity policy strategies are needed to improve activity levels in Canadian youth [34]. In 2005, the Government of Alberta mandated daily physical activity (DPA) to “ensure that all students in grades 1 to 9 are physically active for a minimum of 30 minutes daily through activities that are organized by the school [35].” A survey conducted by Alberta Education to monitor the DPA policy found that, of the surveyed schools, 30% offered physical education everyday prior to 2005 and this number increased to 70% by 2008; the survey did not examine rural and urban differences in DPA implementation [36]. It is therefore surprising that the physical activity levels of urban students remained unchanged during this period, and below what is considered a moderate level of activity, despite government policy aimed at increasing activity levels. This discrepancy may be due to the natural variation in the timing and extent to which DPA is implemented across schools [37], suggesting that additional support and emphasis on facilitating the implementation of physical activity policy are likely needed to increase the activity levels of Alberta youth [16]. Increasing stakeholder engagement could increase the success of physical activity policies such as the DPA by improving their implementation and monitoring [34]. Ensuring that school principals promote the importance of physical activity in addition to academic achievement is likely to result in greater uptake of the physical activity policies.

It is important to note that there were fewer adolescents classified as obese in 2008 compared to 2005, suggesting that the prevalence of obesity in youth may be declining in Alberta. Of note, is the increase in the percentage of rural youth classified as normal weight. Many studies have demonstrated a relationship between child and adolescent obesity and geographical location in Canada and United States see [9, 11–14] and [38]. More specifically, the CCHS found higher levels of overweight/obesity in rural Alberta youth [14]. In this study, differences in the weight status of rural and urban residents were evident in 2005, however, there were no differences in weight status based on geographical location in 2008. It is possible that the increases in physical activity, coupled with a decrease in energy intakes during the period between 2005 and 2008, may have had an impact on weight status.

A limitation of this study includes the cross-sectional design and the use of self-reported survey data. Although there is the potential for recall and response bias with self-reports, the anonymous nature of the survey may have increased the accuracy of reporting. Moreover, this study employed validated measures to assess physical activity and dietary intake. Although we used slightly different recruitment strategies in 2005 as compared to 2008, it is unlikely that this had an effect on the study results. Participant demographics were comparable in both study years. Moreover, in an attempt to account for confounding factors, our analyses controlled for clustering at the school level, SES, age, and sex.

## 5. Conclusions

Several differences in the lifestyle behaviours of adolescents living in rural and urban areas were found in this study, emphasizing the importance of making policy and program recommendations to support healthy lifestyle behaviours within the context of the environments in which adolescents live. Ensuring that healthy lifestyle policies in schools are fully implemented will be necessary to maximise the potential of policy interventions to improve dietary intakes and increase physical activity levels.

## Figures and Tables

**Table 1:** Restricted maximum likelihood (REML) estimates of energy, macronutrient and fibre intakes of rural and urban Alberta students in 2005 and 2008 adjusted for SES, age, and sex.

	2005		2008		*P*-Value
Energy, macronutrients, and fibre*	Rural (*n* = 2712)	Urban (*n* = 2145)	Rural (*n* = 1055)	Urban (*n* = 4059)	
Energy (kcal)	2011 (1945, 2077)	2097 (2020, 2175)	2001 (1910, 2093)	1975 (1912,2039)	.041^†^
Fat (%kcal)	31.7 (31.2, 32.2)	31.7 (31.1, 32.3)	31.2 (30.5, 31.9)	30.3 (29.8,30.7)	.002^†^
Saturated fat (%kcal)	10.8 (10.6, 11.0)	10.7^a^(10.5,10.9)	11.0^b^ (10.7, 11.3)	10.4^c^(10.2, 10.6)	.003^‡^; .033^ac^; <.001^bc^
Carbohydrate (%kcal)	54.6 (54.0, 55.3)	54.8 (54.1, 55.5)	54.6 (53.7, 55.5)	55.4 (54.8, 56.0)	>.05^†^; >.05^‡^
Protein (%kcal)	15.3 (15.1, 15.6)	15.2 (14.9, 15.5)	15.9 (15.6, 16.3)	16.1 (15.9, 16.3)	<.001^†^
Fibre (g)	14.0 (13.4, 14.7)	14.7 (14.0, 15.4)	14.7 (13.9, 15.6)	15.4 (14.8, 15.9)	.03^†^; .042^‡^

*Reported as mean and 95% Confidence Intervals.

^†^Main effect for Year (2005 versus 2008).

^‡^Main effect for (Rural versus Urban).

As a followup to significant interaction superscripts in each row indicate source of significant differences at *P* < .05 using a test of simple effects.

**Table 2:** The diet quality and physical activity levels of rural and urban students in 2005 and 2008.

	2005		2008		*P*-value
	Rural (*n* = 2685)	Urban (*n* = 2185)	Rural (*n* = 1052)	Urban (*n* = 4033)	
	Diet quality*				
Poor^†^	1411 (52.6)^a^	1041 (47.6)^b^	542 (51.5)	2049 (50.8)^c^	<.001^ab^; .016^bc^
Average	1166 (43.4)^a^	1038 (47.5)^b^	461 (43.8)	1803 (44.7)^c^	.005^ab^; .036^bc^
Superior	108 (4.0)	106 (4.9)	49 (4.7)	181 (4.5)	All >.05
	PAQ-C^‡§^				
PAQ-c Score^*||*^	2.87 ± .03^a^	2.90 ± .03	3.00 ± .04^b^	2.88 ± .03^c^	.003^ab^; .008^bc^

*Diet quality is based on whether participants met minimum food group recommendations for Eating Well With Canada's Food Guide and was classified as poor (0-1 food group), average (2-3 food groups), or superior (all 4 food groups).

^†^Reported as *n* (%), superscripts in each row indicate source of significant differences.

^‡^PAQ-C: Scores range from 1 (no physical activity) to 5 (high amount of physical activity) and are adjusted for SES, sex and age. A score of 3 is considerate moderate activity.

^§^Sample size for PAQ-C: (rural 2005: *n* = 2423, urban 2005: *n* = 1938, rural 2008: *n* = 976, urban 2008: *n* = 3097), significant main effect for year (*P* = .024) and an interaction (*P* = .033).

^*||*^Reported as Mean ± standard error. As a follow-up to significant interactions superscripts in each row indicate source of significant differences at *P* < .05 using a test of simple effects to compare differences by year and by residence.

**Table 3:** The weight status of rural and urban students in 2005 and 2008.

	2005		2008		*P*-value
Weight Status^∗^	Rural (*n* = 2314)	Urban (*n* = 1812)	Rural (*n* = 849)	Urban (*n* = 3000)	
Normal weight^†^	1791 (77.4)^a^	1466 (80.9)^b^	690 (81.3)^c^	2453 (81.8)	.007^ab^; .021^ac^
Overweight	377 (16.3)^a^	241 (13.3)^b^	118 (13.9)	418 (13.9)	.007^ab^;
Obese	146 (6.3)	105 (5.8)^a^	41 (4.8)	129 (4.3)^b^	.021^ab^

^∗^International Obesity Task Force cut-offs used to determine weight status.

^†^ Reported as *n* (%), superscripts in each row indicate source of significant differences.

Different subscripts in each row indicate significant differences at *P* < .05 (2-tailed) to compare differences by year and by residence.

## References

[1] Shields M (2006). Overweight and obesity among children and youth. *Health Reports*.

[2] Wang Y, Lobstein T (2006). Worldwide trends in childhood overweight and obesity. *International Journal of Pediatric Obesity*.

[3] Stamatakis E, Zaninotto P, Falaschetti E, Mindell J, Head J (2010). Time trends in childhood and adolescent obesity in England from 1995 to 2007 and projections of prevalence to 2015. *Journal of Epidemiology and Community Health*.

[4] Ogden CL, Flegal KM, Carroll MD, Johnson CL (2002). Prevalence and trends in overweight among US children and adolescents, 1999-2000. *Journal of the American Medical Association*.

[5] Wang Y, Monteiro C, Popkin BM (2002). Trends of obesity and underweight in older children and adolescents in the United States, Brazil, China, and Russia. *American Journal of Clinical Nutrition*.

[6] The Standing Senate Committee on Social Affairs, Science and Technology A healthy, productive Canada: a determinants of health approach. http://www.parl.gc.ca/40/2/parlbus/commbus/senate/com-e/popu-e/rep-e/rephealthjun09-e.pdf.

[7] Tsimeas PD, Tsiokanos AL, Koutedakis Y, Tsigilis N, Kellis S (2005). Does living in urban or rural settings affect aspects of physical fitness in children? An allometric approach. *British Journal of Sports Medicine*.

[8] Townshend T, Lake AA (2009). Obesogenic urban form: theory, policy and practice. *Health and Place*.

[9] Lutfiyya MN, Lipsky MS, Wisdom-Behounek J, Inpanbutr-Martinkus M (2007). Is rural residency a risk factor for overweight and obesity for U.S. children?. *Obesity*.

[10] Willms JD, Tremblay MS, Katzmarzyk PT (2003). Geographic and demographic variation in the prevalence of overweight canadian children. *Obesity Research*.

[11] Plotnikoff RC, Bercovitz K, Loucaides CA (2004). Physical activity, smoking, and obesity among Canadian school youth: comparison between urban and rural schools. *Canadian Journal of Public Health*.

[12] Bruner MW, Lawson J, Pickett W, Boyce W, Janssen I (2008). Rural Canadian adolescents are more likely to be obese compared with urban adolescents. *International Journal of Pediatric Obesity*.

[13] Mitura V, Bollman R (2004). Health status and behaviours of Canada’s youth: a rural-urban comparison. *Rural and Small Town Canada Analysis Bulletin*.

[14] Shields M, Tjepkema M (2006). Regional differences in obesity. *Health Reports*.

[15] Minaker LM, McCargar L, Lambraki I (2006). School region socio-economic status and geographic locale is associated with food behaviour of Ontario and Alberta adolescents. *Canadian Journal of Public Health*.

[16] Loucaides CA, Plotnikoff RC, Bercovitz K (2007). Differences in the correlates of physical activity between urban and rural Canadian youth. *Journal of School Health*.

[17] Veugelers PJ, Schwartz ME (2010). Comprehensive school health in Canada. *Canadian Journal of Public Health*.

[18] Storey KE, Forbes LE, Fraser SN (2009). Diet quality, nutrition and physical activity among adolescents: the Web-SPAN (Web-Survey of Physical Activity and Nutrition) project. *Public Health Nutrition*.

[19] Statistics Canada Education, Skills and Learning Research Papers. Understanding the rural-urban reading gap. http://www.statcan.gc.ca/pub/81-595-m/81-595-m2002001-eng.pdf.

[20] Health Canada Eating Well with Canada’s Food Guide. http://www.hc-sc.gc.ca/fn-an/food-guide-aliment/index-eng.php.

[21] Kowalski KC, Crocker PRE, Faulkner RA (1997). Validation of the Physical Activity Questionnaire for Older Children. *Pediatric Exercise Science*.

[22] Cole TJ, Bellizzi MC, Flegal KM, Dietz WH (2000). Establishing a standard definition for child overweight and obesity worldwide: international survey. *British Medical Journal*.

[23] Schwartz MB, Novak SA, Fiore SS (2009). The impact of removing snacks of low nutritional value from middle schools. *Health Education and Behavior*.

[24] United States Department of Agriculture (2004). *National School Lunch Program*.

[25] Brownell KD, Schwartz MB, Puhl RM, Henderson KE, Harris JL (2009). The need for bold action to prevent adolescent obesity. *Journal of Adolescent Health*.

[26] Jeffery B, Leo A Are schools making the grade? School Nutrition Policies Across Canada. http://cspinet.org/canada/pdf/makingthegrade_1007.pdf.

[27] Dwyer J (2006). Starting down the right path: nutrition connections with chronic diseases of later life. *American Journal of Clinical Nutrition*.

[28] World Health Organisation Diet, nutrition and the prevention of chronic disease. http://www.who.int/hpr/NPH/docs/who_fao_expert_report.pdf.

[29] Simen-Kapeu A, Kuhle S, Veugelers PJ (2010). Geographic differences in childhood overweight, physical activity, nutrition and neighbourhood facilities: implications for prevention. *Canadian Journal of Public Health*.

[30] Bitto EA, Morton LW, Oakland MJ (2003). Grocery stores access patterns in rural food. *Journal for the Study of Food and Society*.

[31] Kaufman PR (1998). Rural poor have less access to supermarkets, large grocery stores. *Rural Development Perspectives*.

[32] Joens-Matre RR, Welk GJ, Calabro MA, Russell DW, Nicklay E, Hensley LD (2008). Rural-urban differences in physical activity, physical fitness, and overweight prevalence of children. *Journal of Rural Health*.

[33] McKenna ML (2010). Policy options to support healthy eating in schools. *Canadian Journal of Public Health*.

[34] Lagarde F, LeBlanc CMA (2010). Policy options to support physical activity in schools. *Canadian Journal of Public Health*.

[35] Bates H (2006). *Daily Physical Activity for Children and Youth: A Review and Synthesis of the Literature*.

[36] Alberta Education (2008). *Daily Physical Activity Survey Report*.

[37] Ramanathan S, Allison KR, Faulkner G, Dwyer JJM (2008). Challenges in assessing the implementation and effectiveness of physical activity and nutrition policy interventions as natural experiments. *Health Promotion International*.

[38] McMurray RG, Harrell JS, Bangdiwala SI, Deng S (1999). Cardiovascular disease risk factors and obesity of rural and urban elementary school children. *Journal of Rural Health*.

